# High prevalence of undocumented SARS-CoV-2 infections revealed by analysis of nucleocapsid-specific IgG responses in diagnosed and undiagnosed individuals

**DOI:** 10.1371/journal.pgph.0003300

**Published:** 2025-01-22

**Authors:** Kim Blom, Ilias Galanis, Philip Bacchus, Klara Sondén, Ioana Bujila, Tatiana Efimova, Fredrik Garli, Mikael Mansjö, Elin Movert, Aleksandra Pettke, Marie Rapp, Maike Sperk, Sandra Söderholm, Karin Valentin Asin, Sarah Zanetti, Magnus Gisslén, Andreas Bråve, Ramona Groenheit, Jonas Klingström

**Affiliations:** 1 Public Health Agency of Sweden, Solna, Sweden; 2 Lund University, Lund, Sweden; 3 Swedish Armed Forces, Umeå, Sweden; 4 University of Gothenburg, Gothenburg, Sweden; 5 Sahlgrenska University Hospital, Gothenburg, Sweden; 6 Linköping University, Linköping, Sweden; University of Cape Town Faculty of Health Sciences, SOUTH AFRICA

## Abstract

Acute SARS-CoV-2 infections are not always diagnosed; hence an unknown proportion of all infections are not documented. SARS-CoV-2 can induce spike and nucleocapsid protein specific IgG antibodies, which can be detected in seroprevalence studies to identify a previous infection. However, with the introduction of vaccines containing the spike protein it is no longer possible to use spike-IgG as a marker of infection. In many countries marketed vaccines do not include the nucleocapsid protein, allowing the use of nucleocapsid-specific IgG (N-IgG) as a specific marker for previous infection. Importantly however, not all SARS-CoV-2-infected individuals develop detectable N-IgG responses and there are reports of waning of N-IgG titers in previously infected individuals, complicating the use of N-IgG in seroprevalence studies. Here, our aim was to investigate N-IgG as a marker for previous infection. To this end we analyzed a well characterized cohort (n = 2,583; sampled in March, 2022), including 612 participants with a previously diagnosed and documented SARS-CoV-2-infection. We show that 75% (460/612) of the confirmed SARS-CoV-2-infected participants were N-IgG positive, and that the frequency of seropositivity was stable for at least 105 weeks after the latest documented SARS-CoV-2-infection. Among participants with no documented SARS-CoV-2-infection, 32.6% (642/1971) were N-IgG-positive, suggesting a previous infection. Assuming similar frequency of N-IgG-seronegative cases in previously diagnosed and undiagnosed individuals we further estimate that 214 of the 1329 undiagnosed and N-IgG-negative cases had been previously infected, indicating a total infection rate of 56.8% (1,468/2,583), clearly higher than the documented 23.7% rate of infection, in this cohort. In conclusion, our results suggest that while N-IgG is a good marker of previous SARS-CoV-2-infection the large proportion of previously infected N-IgG-negative individuals introduces a risk for underestimations of total level of previously infected individuals in a population. Accounting for this dark number of undiagnosable cases can provide better estimates of total level of infected individuals in a population.

## Introduction

SARS-CoV-2 causes COVID-19 and can be detected during the acute phase of the disease, allowing for reporting of documented diagnosed cases. However, all acute SARS-CoV-2 infections are not diagnosed, and hence not documented in health registers [1,2]. These undocumented cases represent a challenge when it comes to estimating total number of previously infected individuals in a population. May 5, 2023 the World Health Organization (WHO) declared the end of COVID-19 as a global health emergency. However, COVID-19 continues to cause global health problems, and SARS-CoV-2 surveillance is therefore still important [3]. SARS-CoV-2 is evolving fast, and new variants may raise and spread globally [4]. To continue monitoring the effects of the pandemic, and effects of counter-measures such as current and future vaccines, serological surveillance will continue to be an important tool [5,6].

SARS-CoV-2 infection typically induces long-lasting specific anti-spike and anti-nucleocapsid protein IgG responses. These antibody responses can be used as markers in seroprevalence studies to identify previously infected individuals. However, also COVID-19 vaccinated individuals develop high levels of spike-specific IgG (S-IgG), indistinguishable from SARS-CoV-2 infection-induced S-IgG. In contrast, the marketed vaccines in most countries, including those available in Sweden, do not include the nucleocapsid protein and therefore do not induce nucleocapsid-specific IgG (N-IgG). This makes N-IgG a suitable specific marker for previous infection, including in highly vaccinated populations [7]. Importantly, while SARS-CoV-2 infection normally induces high N-IgG levels, N-IgG is not detected in all previously infected individuals [8,9]. These individuals represent a group of undocumented infections that cannot be identified in seroprevalence studies, complicating the attempts to use N-IgG seroprevalence as a measure of total level of previous infections in a population. To estimate the size of this group of previously SARS-CoV-2-infected seronegative individuals is a challenge.

The objective of this study was to better understand the total prevalence of SARS-COV-2-infected individuals by estimating the “dark number” of previously infected anti-N-IgG negative individuals. To this end, we analyzed the SARS-CoV-2 N-IgG status in combination with registry data of earlier documented diagnosed acute infections in a well characterized Swedish cohort with 2,583 participants, sampled in March 2022 [10]. Based on these data, we calculated overall infection rate and estimated the level of undocumented infections in this cohort, and finally estimated the total level of infections in the Swedish population.

## Materials and methods

### Study design

This study is based on samples and data from a survey performed in Sweden, March 21–25, 2022 [10]. Individuals in a nationwide probability-based web panel [1,11] regularly used for health-related questionnaires at the Public Health Agency of Sweden were invited to participate. Participants enrolled by signing up and then provided informed consent. In total 11,334 individuals from the web panel were invited, of them 2,906 signed up to the study. The age span was 2–96 years and included participants from all 21 regions of Sweden. For those below 16 years of age, the legal guardian provided consent. After enrolling, the participants received, via regular post, self-sampling material for use at home and written instructions on how to use them. Participation could be withdrawn at any time-point before or after the sampling.

### Blood sampling

The cohort, self-sampling procedure, collection, and extraction of samples have previously been described [1,10,12,13]. Briefly, participants provided dry blood spots (DBS) by performing blood self-sampling by fingerpicking followed by collection of blood on a qDBS (Capitainer) as previously described [10,12,13]. For participants under the age of 16, the blood sampling was performed by a caregiver.

### Serological assay

Samples were extracted from the qDBS as previously described [12,13]. N-IgG responses were then analysed using MSD V-PLEX SARS-CoV-2 Panel 2 (IgG) kit (Meso Scale Diagnostics), according to the manufacturer’s instructions. The WHO international standard for anti-SARS-CoV-2 immunoglobulin was used to translate results into Binding Antibody Units (BAU)/ml [14]. Data from participants with a valid serological response (n = 2,583, four of the 2,587 samples could not be properly processed) were further analysed. The cut-off for N-IgG was 11.8 BAU/ml, samples with titers above the cut-off were considered as positive (Meso Scale Diagnostics). The specified specificity of the test is 100% (Meso Scale Diagnostics).

### Register data

Register data on previously confirmed reported infections were available from the Swedish national registry for notifiable diseases (SmiNet).

### Statistical analyses

We estimated the number of SARS-CoV-2-infected individuals among those without a documented infection in the Swedish population (N_INF,ND_) until March 2022, with the use of parametric bootstrap [15] based on the binomial distribution. Ten thousand samples were drawn, by age group (2–11, 12–19, 20–29, 30–49, 50–64, 65–79, ≥80), from both groups of documented SARS-CoV-2-infected individuals and those without a documented infection. For each bootstrap sample, the estimated proportion of nucleocapsid-IgG (N-IgG) positive among the non-documented individuals (P_POS,ND_) was divided by the estimated proportion of nucleocapsid-IgG (N-IgG) positive among those with a documented infection (P_POS,D_), in order to account for the undiagnosable infections among the undocumented cases. It was ensured that the result of this division could not exceed 100%. The resulting proportion was then multiplied by the population size of the respective age group and the estimates from all age groups were added together. For one bootstrap iteration the estimation formula is the following:


$$N_{INF,ND}\;=\;\overset{7}{\underset{i=1}{\sum }}\left(P_{POS,ND\;age\;group\;\left(i\right)}/P_{POS,D\;age\;group\;\left(i\right)}\right)*\left(Pop.size_{ND\;age\;group\;\left(i\right)}\right)$$

The 2.5 and 97.5 percentiles of the ten thousand bootstrap estimates were used to obtain the 95% confidence interval. The number of known documented PCR-confirmed SARS-CoV-2 cases was finally added to estimate the total number of SARS-CoV-2-infected individuals in the Swedish population. Analyses were performed in R (https://www.r-project.org).

### Ethics statement

This study was performed as part of the Public Health Agency of Sweden’s assignment to monitor communicable diseases and evaluate infection control measures in accordance with §§18 of the ordinance (2021:248) from the Swedish Parliament. All participants provided informed consent online. For those under 16 years of age, the legal guardian was asked to, and provided, informed consent.

## Results

By late March 2022, 612 (23.7%) of the 2,583 participants in the cohort had had at least one reported documented SARS-CoV-2 infection (Table 1). This is similar to the overall frequency of infections in Sweden by that time point: 23% of the total Swedish population had had at least one reported documented SARS-CoV-2 infection (Table 1). While the overall frequencies of previously reported SARS-CoV-2 infections were similar in our cohort and the Swedish population, we noted higher frequency of reported infections in our cohort’s youngest age group and lower frequency of reported infections in the oldest age group (Table 1).

**Table 1:** Summary of documented SARS-CoV-2 infections among the 2,583 participants and in the Swedish population, March, 2022.

Stratification	Cohort		Total Swedish population
	Total, no.	Registered infections*, no. (%)	Registered infections* (%)
Overall	2583	612 (23.7)	23.0
Age, years			
2–11	113	34 (30.1)	16.6
12–19	64	17 (26.6)	27.1
20–29	175	53 (30.3)	29.8
30–49	760	245 (32.2)	32.0
50–64	745	182 (24.4)	23.3
65–79	637	76 (11.9)	8.3
>80	89	5 (5.6)	11.0

*Reported in SmiNet, the national notification system for communicable diseases.

**Fig 1:**
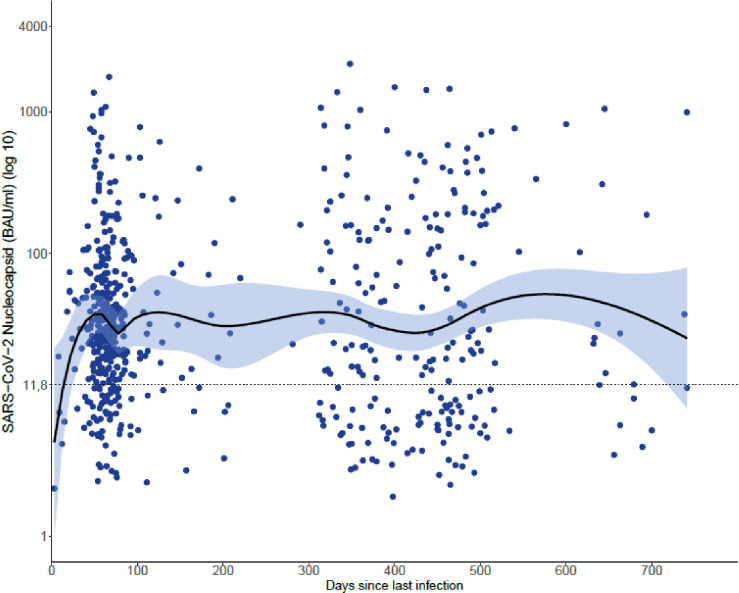
No general antibody waning over time in individuals with a previous confirmed SARS-CoV-2 infection. Data shown represent N-IgG titers over time after the last documented infection in the 612 confirmed SARS-CoV-2 infected individuals. The N-IgG titers represent Binding Antibody Units (BAU)/ml. Cut-off for a positive response is 11.8 BAU/ml. LOESS curve; the shaded area represents 95% confidence interval.

We first analyzed N-IgG responses in the 612 participants with a previously reported infection. While 75.2% (460/612) of them were positive for N-IgG, in 24.8% (152/612) of the samples N-IgG was not detected (Table 2, Fig 1), showing that almost 25% of those with a documented earlier infection would be unaccounted for in a seroprevalence survey not cross-linked to clinical data.

**Table 2:** Summary of SARS-CoV-2 N-IgG data among the 612 participants with a confirmed reported infection.

Stratification	N-IgG test, no (%)	
	Positive	Negative
Overall	460 (75.2)	152 (24.8)
Age, years		
2–11	28 (82.4)	6 (17.6)
12–19	17 (100)	0 (0)
20–29	43 (81.1)	10 (18.9)
30–49	191 (78.0)	54 (22.0)
50–64	124 (68.1)	58 (31.9)
65–79	53 (69.7)	23 (30.3)
≥80	4 (80.0)	1 (20.0)

There are conflicting data regarding time kinetics of N-IgG; some have reported short, and others long, durability [16–22]. For this cohort we observed that the frequency of N-IgG positive responses over time in the 460 N-IgG-positive participants was rather stable up to at least 105 weeks after the last reported infection (Fig 1). Frequency of previously infected and N-IgG-positive participants varied between age groups (Table 2).

**Fig 2:**
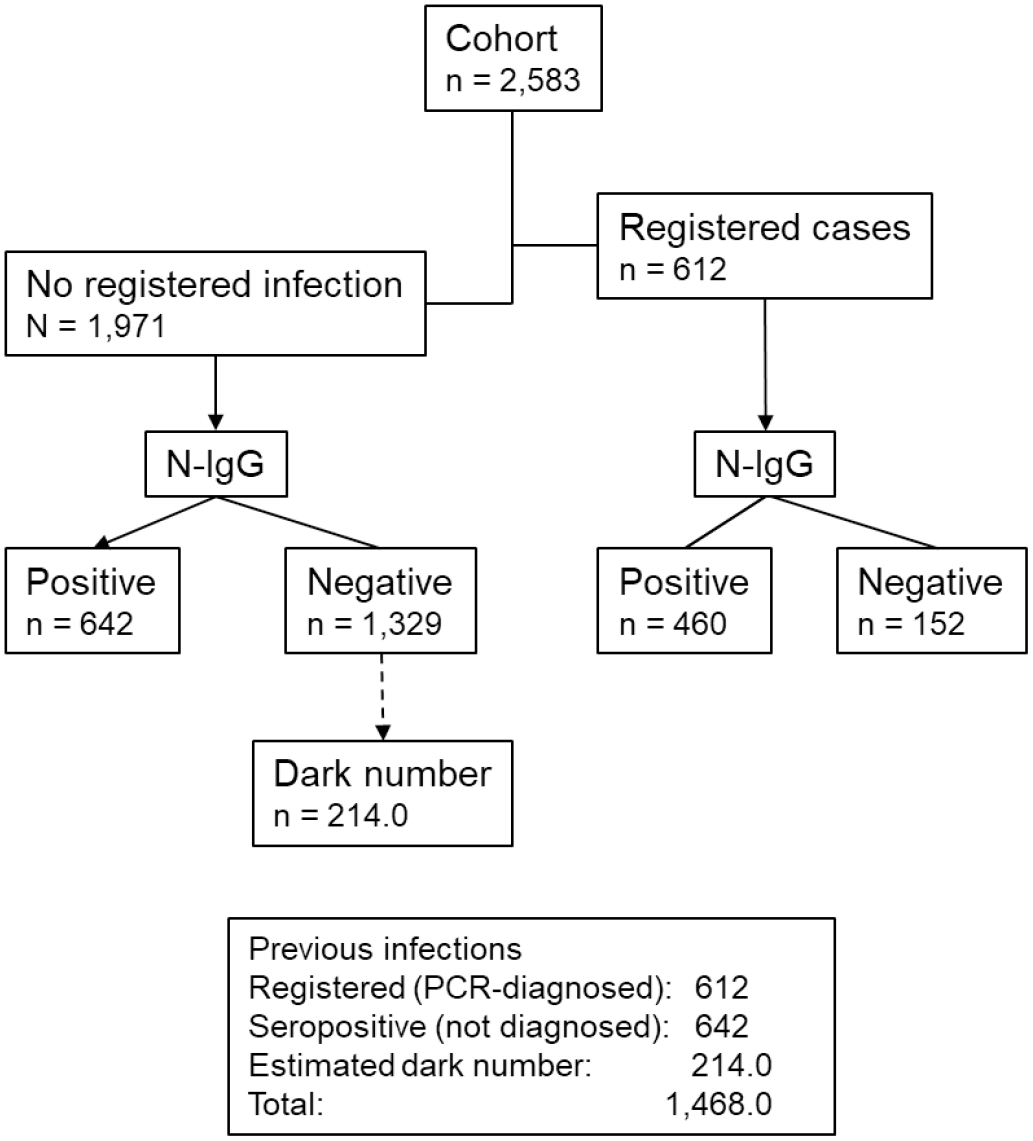
Flow chart showing study design and results. Dark number; undocumented and undiagnosable infections.

We next analyzed the N-IgG responses in the group of 1,971 participants with no previous registered SARS-CoV-2 infection. In this group, 32.6% (642/1971) were N-IgG positive (Fig 2, S1 Table), suggesting a high level of previous undocumented SARS-CoV-2 infections. The frequency of N-IgG positive participants with no previous registered SARS-CoV-2 infection differed in the different age groups, ranging from 69.6% in 2–11 years old to 14.3% in those ≥80 years (S1 Table).

When combining the group with reported documented SARS-CoV-2 infections (n = 612), with the additionally identified 642 participants that had seroconverted (positive for N-IgG) in the group with no previous reported SARS-CoV-2 infection (Fig 2), the total level of previously infected participants in this cohort increased from 23.7% to 48.5%.

We next estimated the number of infections in the group of N-IgG-negative participants with no documented previous infection (“dark number”; undocumented and serologically undiagnosable cases). To this end, we assumed the frequency of previously infected and N-IgG-negative cases to be similar among both diagnosed and undiagnosed individuals in the cohort. Based on this assumption, we estimated that 214 additional participants in this cohort might have been previously infected (Fig 2, S1 Table). This would indicate a total number of 1,468 (56.8%) previously infected participants in the cohort (Table 3, Fig 2), more than twice than those 612 identified by clinical data alone, and 33% higher than the 1,102 identified by N-IgG serology alone.

**Table 3:** Estimations of total amount of previous SARS-CoV-2 infections among the 2,583 participants.

Stratification	Participants, No.	Documented infections	Undocumented infections		Total estimated infected, No. (%)
		PCR-positive, No.	N-IgG positive, No.	N-IgG negative, estimated No.	
Overall	2,583	612	642	214.0	1,468.0 (56.8)
Age, years					
2–11	113	34	55	11.8	100.8 (89.2)
12–19	64	17	23	0	40.0 (62.5)
20–29	175	53	46	10.7	109.7 (62.7)
30–49	760	245	239	67.6	551.6 (72.6)
50–64	745	182	151	70.6	403.6 (54.2)
65–79	637	76	116	50.3	242.3 (38.0)
≥80	89	5	12	3.0	20 (22.5)

By March 2022, 23% of the Swedish population (2350106/10222613) had been diagnosed with at least one SARS-CoV-2 infection. Based on the results from our cohort, we estimate that in addition 48.6% (95% CI: 45.0–52.7; n = 3824751, 95% CI: 3542912–4147761) of the 7872507 Swedes with no previously confirmed infection had been infected, suggesting that totally 60.4% (95% CI: 57.7–63.6) of the population had been infected at least once until March 2022 (Table 4).

**Table 4:** Estimated total incidence of SARS-CoV-2 infected individuals in Sweden, March 2022 (overall population in Sweden, March 2022: 10222613).

Stratification	Documented SARS-CoV-2 infection, no. (%)		No documented SARS-CoV-2 infections		Total estimated infections	
	yes	no	Estimated undocumented infection, no. (%)	95% CI no. (%)	Estimated total infected, No. (%)	95% CI no. (%)
Population	2350106 (23.0)	7872507 (77.0)	3824751 (48.6)	3542912–4147761 (45.0–52.7)	6174857 (60.4)	5893018–6497867 (57.7–63.6)

## Discussion

Combining registry data with serological data in a well characterized cohort, our results suggest that there is a considerable number of previous undiagnosed SARS-CoV-2 infections that are not possible to detect in seroprevalence studies.

Underestimations of the total level of previous SARS-CoV-2-infections can be attributed to at least two factors; a biological factor reflecting that not all who have had a SARS-CoV-2 infection are positive for N-IgG, and a societal factor, reflecting the under-reporting of acute infections due to limited testing of COVID-19 cases, especially of mild/asymptomatic infections. There are conflicting reports regarding waning of N-IgG levels, and if frequency of N-IgG-positive cases decreases with time since infection. While some have reported rapid waning [16,17], others report rather stable N-IgG levels over time [7,18–22]. In this cohort, we observed a rather stable frequency of individuals with detectable, positive, N-IgG levels for up to at least 105 weeks after the latest known infection. In some individuals undiagnosed reinfection might contribute to them maintaining high titers over time. Although N-IgG-titers will ultimately decrease over time, titers early after infection are often more than 30-fold higher than the cut-off for a positive response [9], suggesting N-IgG is a suitable biomarker for detection of previous SARS-CoV-2 infection. Importantly however, not all previously infected individuals are positive for N-IgG [8,9], which can result in a general underestimation of previous infections in serological screenings.

An important aspect of SARS-CoV-2 seroprevalence studies is the assay used for detection and/or quantification of specific antibody responses [8]. Collection of blood on DBS simplifies seroprevalence studies [12,23,24], allowing for studies of large cohorts without the need for collecting and transporting serum. The serological assay used in this study has been validated [23], and has earlier also been used in studies of DBS samples [25]. It has further been used in seroprevalence studies in Sweden [10]. Importantly, many SARS-CoV-2 infected individuals do not produce detectable levels of N-IgG [8,9], and they can therefore not be identified as previously infected based on N-IgG responses alone. In line with this, we could not detect a positive N-IgG response in 25% of previously infected participants.

Based on the findings in this cohort we estimate that 60.4% of the Swedish population had been infected with SARS-CoV-2 before end of March 2022. While there are several unknowns, this indicates that more than half of Swedish population had, at this time-point, been infected at least once with SARS-CoV-2.

There are some limitations in this study. Participation bias has resulted in having a higher proportion of females and individuals with higher education in the study compared to the Swedish population. SARS-CoV-2 infection rates have been shown to be similar in males and females [26], suggesting that the higher frequency of females has a small impact on the overall results. The discrepancy in education is likely to have induced some bias but this effect should be mitigated by the fact that the study takes place two years after the start of the pandemic. Finally, these data are from March 2022, later N-IgG responses induced by Omicron might have changed the frequency of N-IgG positive infected individuals.

To summarize, the result show that a large part of previously SARS-CoV-2-infected individuals that were not diagnosed during acute infection are N-IgG negative, indicating that N-IgG based seroprevalence studies risk underestimating the total level of infections.

## Conclusions

A proportion of previously SARS-CoV-2 infected individuals do not produce detectable levels of N-IgG. If this is not accounted for, seroprevalence studies will underestimate the total level of previously infected individuals. Seroprevalence studies including data from high quality registers of documented diagnosed infections can allow for estimations of total level of previous infections in a cohort, and can provide estimations of frequencies of undocumented infected individuals.

## Supporting information

S1 Table Summary of testing status of SARS-CoV-2 N-IgG and estimated numbers of infections among 1971 participants with no previous documented infection. (DOCX)
