# Supplementary material for: High prevalence of undocumented SARS-CoV-2 infections revealed by analysis of nucleocapsid-specific IgG responses in diagnosed and undiagnosed individuals
Source: PLOS Glob Public Health. 2025 Jan 22;5(1):e0003300. doi: 10.1371/journal.pgph.0003300 (PMC11753678; doi:10.1371/journal.pgph.0003300)
Supplement: S1 Table — (DOCX) [file pgph.0003300.s001.docx]

**Supplementary material**

**High prevalence of undocumented SARS-CoV-2 infections revealed by analysis of**

**nucleocapsid-specific IgG responses in diagnosed and undiagnosed individuals**

Kim Blom^1^, Ilias Galanis^1¶^, Philip Bacchus^2,3¶^, Klara Sondén^1^, Ioana Bujila^1^, Tatiana Efimova^1^, Fredrik Garli^1^, Mikael Mansjö^1^, Elin Movert^1^, Aleksandra Pettke^1^, Marie Rapp^1^, Maike Sperk^1^, Sandra Söderholm^1^, Karin Valentin Asin^1^, Sarah Zanetti^1^, Magnus Gisslén^1,4,5^, Andreas Bråve^1^, Ramona Groenheit^1&^, and Jonas Klingström^1,5&*^

^1^ Public Health Agency of Sweden, Solna, Sweden

^2^ Lund University, Lund, Sweden

^3^ Swedish Armed Forces, Umeå, Sverige

^4^ University of Gothenburg, Gothenburg, Sweden

^5^ Sahlgrenska University Hospital, Gothenburg, Sweden

^6^ Linköping University, Linköping, Sweden

**S1 Table.** Summary of testing status of SARS-CoV-2 N-IgG and estimated numbers of infections among 1971 participants with no previous documented infection.

|  | N-IgG test, no (%) | |  |  |
| --- | --- | --- | --- | --- |
| Stratification | Positive | Negative | Estimated infected N-IgG negative, No. | Total estimated infected, No. (%) |
| Overall | 642 (32.6) | 1329 (67.4) | 214.0 | 856.0 (43.3) |
| Age, y |  |  |  |  |
| 2–11 | 55 (69.6) | 24 (30.4) | 11.8 | 66.8 (84.5) |
| 12–19 | 23 (48.9) | 24 (51.1) | 0 | 23.0 (48.9) |
| 20–29 | 46 (37.7) | 76 (62.3) | 10.7 | 56.7 (46.5) |
| 30–49 | 239 (46.4) | 276 (53.6) | 67.6 | 306.6 (59.5) |
| 50–64 | 151 (26.8) | 412 (73.2) | 70.6 | 221.6 (39.3) |
| 65–79 | 116 (20.7) | 445 (79.3) | 50.3 | 166.3 (29.7) |
| >80 | 12 (14.3) | 72 (85.7) | 3.0 | 15.0 (17.9) |
